# Gene expression hallmarks of cellular ageing

**DOI:** 10.1007/s10522-018-9750-z

**Published:** 2018-02-28

**Authors:** Stephen Frenk, Jonathan Houseley

**Affiliations:** 1 Department of Genetics, University of North Carolina, Chapel Hill, NC 27599-3280 USA; 2 Epigenetics Programme, Babraham Institute, Cambridge, UK

**Keywords:** Ageing, Aging, Gene expression, Transcriptome, Hallmark, Clock

## Abstract

Ageing leads to dramatic changes in the physiology of many different tissues resulting in a spectrum of pathology. Nonetheless, many lines of evidence suggest that ageing is driven by highly conserved cell intrinsic processes, and a set of unifying hallmarks of ageing has been defined. Here, we survey reports of age-linked changes in basal gene expression across eukaryotes from yeast to human and identify six gene expression hallmarks of cellular ageing: downregulation of genes encoding mitochondrial proteins; downregulation of the protein synthesis machinery; dysregulation of immune system genes; reduced growth factor signalling; constitutive responses to stress and DNA damage; dysregulation of gene expression and mRNA processing. These encompass widely reported features of ageing such as increased senescence and inflammation, reduced electron transport chain activity and reduced ribosome synthesis, but also reveal a surprising lack of gene expression responses to known age-linked cellular stresses. We discuss how the existence of conserved transcriptomic hallmarks relates to genome-wide epigenetic differences underlying ageing clocks, and how the changing transcriptome results in proteomic alterations where data is available and to variations in cell physiology characteristic of ageing. Identification of gene expression events that occur during ageing across distant organisms should be informative as to conserved underlying mechanisms of ageing, and provide additional biomarkers to assess the effects of diet and other environmental factors on the rate of ageing.

## Introduction

The ageing process encompasses progressive dysfunction in almost every organ in almost every organism, forming an immense challenge to modern medicine that seems to require many parallel interventions addressing an increasingly complex series of age-related conditions. A set of ‘Hallmarks of ageing’ have been defined, providing a framework for understanding ageing pathologies at the cellular and organismal level, but how these hallmarks arise and how cells respond to them often remains unclear (Lopez-Otin et al. 2013). Given the complexity of the process, it is not too surprising that an array of studies of the ageing transcriptome have been undertaken over the past two decades attempting to shed light on the underlying drivers of pathology. These studies have spanned organisms from yeast to man, taking advantage of the remarkable conservation of ageing pathology across eukaryotes to examine the ageing process in experimentally tractable model organisms. However, major challenges have been encountered in the performance of reproducible experiments and the extraction of meaningful data; early studies particularly in higher eukaryotes found few changes in common, and it is only with more recent meta-analyses of giant cross-sectional datasets that a consensus is starting to emerge on a set of gene expression changes that are associated with ageing. Here we survey the literature of transcriptomic changes associated with normal ageing across evolutionarily diverse eukaryotes and describe a set of potential gene expression hallmarks indicative of underlying age-linked gene expression programmes.

Do underlying age-linked gene expression programmes exist? On one hand, it is tempting to suggest that age-linked changes in gene expression reflect noise, caused by the accumulation of random mutations and epigenetic changes, leading to aberrant activation or repression of promoters. Age-linked accumulation of DNA damage has been detected in human brain samples and demonstrated to reduce gene expression (Lu et al. 2004; O’Hagan et al. 2008), perhaps an unsurprising result given the endogenous rate of DNA damage (De Bont and van Larebeke 2004). Nonetheless, age-linked gene expression changes cannot be purely ascribed to damage in all organisms. Firstly, such changes clearly occur in budding yeast (Hu et al. 2014; Kamei et al. 2014; Lesur and Campbell 2004; Yiu et al. 2008) in which the rate of age-linked promoter mutation is negligible (Kaya et al. 2015). Secondly, particular categories of genes reproducibly change in expression with age across a wide range of organisms, an observation that is very hard to reconcile with direct effects of random promoter damage.

Age-linked gene expression programmes could arise for many reasons and do not necessarily equate to programmed ageing. Gene expression changes can be reactive to alterations in cellular and organismal physiology, and if those alterations reproducibly occur during ageing then the responsive events form an age-associated gene expression programme; a response to accumulating DNA damage would be an example of this. More subtly, organisms may have evolved specific gene expression programmes to mitigate degenerative processes associated with ageing as long as these programmes extend reproductive lifespan or fecundity. The common model organisms used by molecular biologists inhabit disparate environments and will certainly have evolved different life history strategies to deal with the interplay between longevity and fecundity, implying that such programmes will display considerable variation across organisms. Conversely, of course, this suggests that any highly conserved aspects of age-associated gene expression represent core signatures of the ageing process and general responses to it.

Gene expression is not the only molecular outcome of ageing. Many pathological changes may have no associated gene expression response, and key signalling and metabolic changes act primarily at the level of translation, post-translational modifications or metabolite abundance, differences that only indirectly affect the gene expression pattern if at all. Furthermore, changes in steady-state mRNA levels as measured in mRNAseq or microarray analyses provide an inexact approximation of transcriptional activity, as discussed in “Additional Information”. Nonetheless, conserved gene expression changes provide a rich dataset in which to probe the pathological processes and evolutionary mechanisms underlying ageing, particularly when gene expression signatures are reproducibly observed across phyla.

## The difficulties in detecting conserved transcriptional signatures of ageing

Studies in humans have often unearthed poorly-overlapping sets of age-associated gene expression signatures between different tissues, and it remains the case that tissue-specific changes tend to be of greater magnitude and therefore most obvious (compare for example Farr et al. 2015; Glass et al. 2013; Khoo et al. 2014; Nakamura et al. 2012; Zahn et al. 2006). These disparities imply that ageing follows a different course in each tissue, rather than comprising a universal molecular programme in diverse tissues. An obvious confounding factor is changing cell composition; for example blood, the most routinely sampled tissue in human studies, changes dramatically with age. Both CD4+ and CD8+ T cell samples from young donors are rich in naïve T cells whereas such populations from old donors contain greater frequencies of memory T cells (reviewed in Arnold et al. 2011). It is highly plausible that increased exposure to infections and other immunological challenges that naturally accompany age provide the major driver for such changes. It is also possible that processes such as homeostatic expansion have independent effects upon the ageing T cell repertoire unrelated to extrinsic antigen exposure. Either way, these pressures on blood cell population composition cause problems when trying to identify cell-intrinsic gene expression changes in T cells across organismal lifespan. Disentangling cell-intrinsic signatures of age-linked gene expression within a cell population of fluid composition therefore requires extensive cell purification, which is often impractical in the large studies required to achieve useful statistical power. Studies of smaller model organisms meet similar problems, for example, profound age-linked differences in gene expression are expected in ageing female flies because they become thinner and stop producing eggs. Although this problem can be avoided by assaying males or individual body parts such as heads, similar problems are likely to be encountered.

A further complication comes from the assumed linearity of age-linked gene expression change, which is widespread and seemingly logical given the progressive nature of the ageing process. Time-course analyses in budding yeast are broadly in accord with this idea (Cruz et al. 2017; Janssens et al. 2015), but studies in worms and flies have revealed a significant number of genes following non-linear trajectories across the life-course including some with their apex or nadir at middle age (Lund et al. 2002; Pletcher et al. 2002). More extreme conclusions have arisen in a number of human cross-sectional studies, which have reported the greatest expression variability at middle age (Gheorghe et al. 2014; Haustead et al. 2016; Remondini et al. 2010), with some aged samples being more closely related to young samples. This may or may not reflect a survivor effect, in other words that the donors of old tissue never underwent the gene expression changes linked with pathology at middle age and therefore survived to old age. Such differences have the potential to be hugely informative as to the order of events and the hierarchy of cause and effect in ageing, but show that simple comparisons of young versus old, as performed in many model organism studies, probably miss important events in the gene expression landscape.

The only real solution to these problems is to search for age-linked gene expression changes that are conserved across tissues and across organisms, and follow clear ageing trajectories. This requires cross species comparisons of gene expression data derived at high resolution across the life course using in vivo samples as opposed to ageing in cell culture. Recent studies have discovered consistent changes in related categories of genes across disparate datasets (see below), though these have required highly replicated sample sets at multiple time points or large cross-sectional meta-analyses to overcome confounding effects. Some very detailed meta-analyses have also directly addressed the question of whether conserved gene expression signatures can be detected across different cell types and organisms, identifying specific genes differentially expressed across datasets (de Magalhaes et al. 2009; Plank et al. 2012; Voutetakis et al. 2015), and on-line resources exist to facilitate investigation of these gene expression signatures (Digital Ageing Atlas; Human Ageing Geomic Resource). However, the subtlety of many of these changes relative to tissue specific differences leaves open the fundamental question of whether general, conserved, cell intrinsic processes actually underlie ageing.

## Cellular ageing clocks

In support of this idea, remarkably accurate epigenetic clocks have been described in humans based on progressive epigenetic changes that occur at the same sites in (almost) all tissues regardless of their private age-linked gene expression programmes (Hannum et al. 2013; Horvath 2013). Clocks have now been found in multiple species, but are of course restricted to those with DNA methylation (Stubbs et al. 2017; Thompson et al. 2017; Wang et al. 2017). Such clocks are remarkably accurate, predicting chronological age to within 3–4 years in humans, but showing expected derivations in individuals with a lower or higher biological age through diet (Horvath et al. 2014; Quach et al. 2017) or medical condition (Horvath et al. 2016; Maierhofer et al. 2017). This suggests that underlying cell-intrinsic processes drive ageing in all tissues at roughly the same rate, and therefore that tissue-independent features of age-associated gene expression patterns are likely to provide insights into the critical underlying drivers of ageing.

Despite the difficulties in detecting weak underlying gene expression signatures, an effective gene expression clock has also been constructed with good predictive value for chronological age in humans (Peters et al. 2015). Additionally, a small number of genes show larger changes with age and can be used to segregate cohorts by age and provide insights into biological age (Harries et al. 2011; Holly et al. 2013b). Unfortunately, there is not a clear relationship between differentially expressed genes in these transcriptional clocks and differentially methylated sites in the epigenetic clocks. Nor in fact is there any strong bias in the function of genes that contain differentially methylated sites. Therefore, the epigenetic clock is in itself unlikely to be the direct driver of ageing, but rather it should be considered that epigenetic and gene expression changes provide parallel readouts of the ageing process.

## Candidates for gene expression hallmarks of cellular ageing

**Fig. 1:**
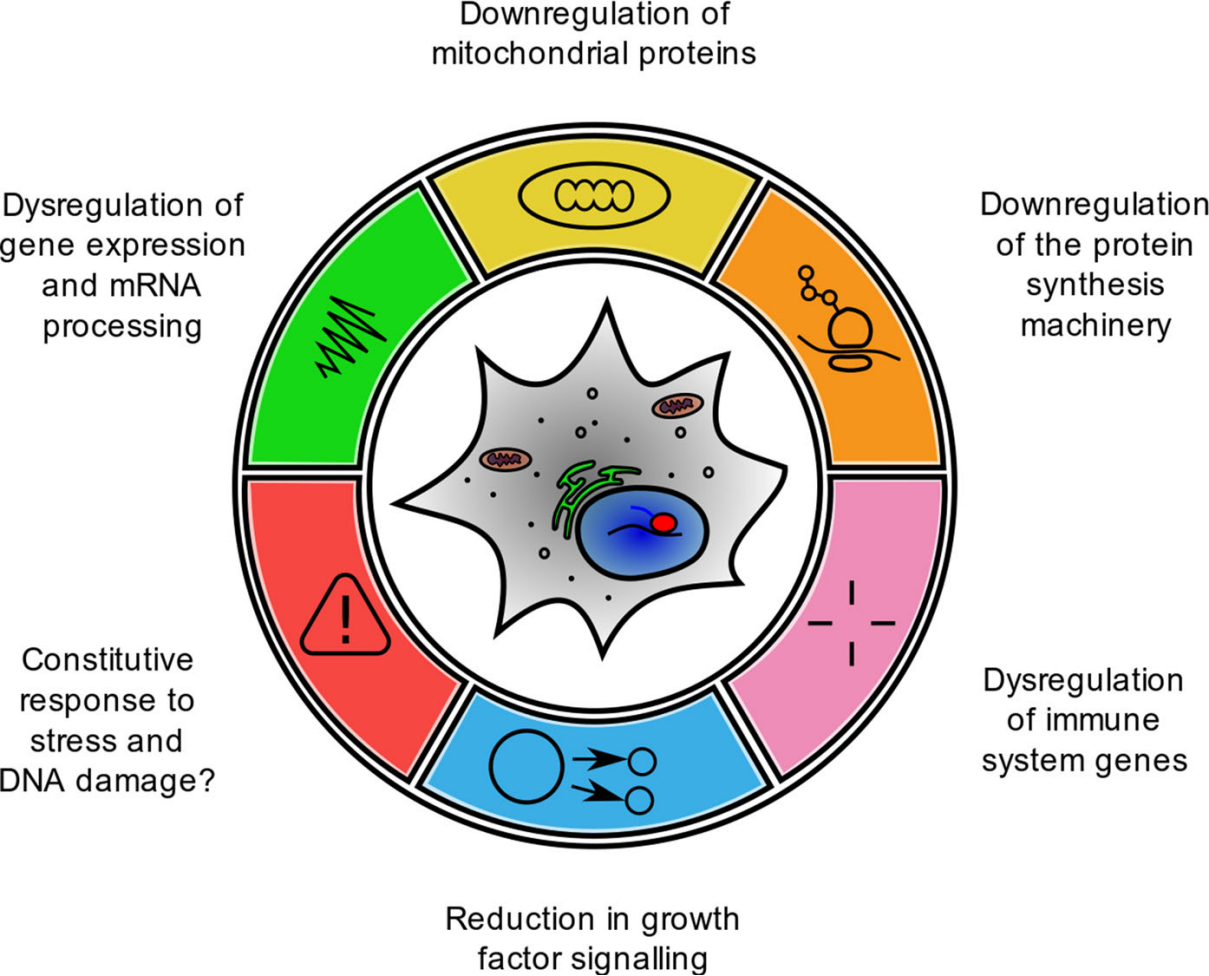
Gene expression hallmarks of cellular ageing

Studies of the ageing transcriptome have been performed in yeast, worms, flies, rodents and humans along with a few non-standard models, over two decades using platforms available at the time. During this period both the signal-to-noise and the cost-per-sample for genome-wide expression analysis has improved considerably and therefore the power of studies has steadily increased. This has been matched by improved analysis methods and richer gene ontologies for functional annotation. We therefore place more weight on modern studies while trying to refer back to earlier reports where informative. Despite these limitations, many consistent age-associated gene expression changes have been reported, and we have classed the various signatures occurring repeatedly across different tissues and organisms into six candidate gene expression hallmarks of cellular ageing (Fig. 1):

1. Downregulation of genes encoding mitochondrial proteins
2. Downregulation of the protein synthesis machinery
3. Dysregulation of immune system genes
4. Reduction in growth factor signalling
5. Constitutive responses to stress and DNA damage
6. Dysregulation of gene expression and mRNA processing

Below, we address the available evidence underlying our choice of these as conserved gene expression hallmarks in the ageing of disparate organisms.

### Downregulation of genes encoding mitochondrial proteins

The change most consistently reported in ageing transcriptome studies is a down-regulation of mitochondrial protein mRNAs, particularly nuclear-encoded components of the electron transport chain (ETC) and mitochondrial ribosomal proteins. This is observed in humans (de Magalhaes et al. 2009; Peters et al. 2015; van den Akker et al. 2014), rodents (de Magalhaes et al. 2009; Zahn et al. 2006, 2007), flies (Cannon et al. 2017; Girardot et al. 2006; Landis et al. 2004; McCarroll et al. 2004; Pletcher et al. 2002; Zahn et al. 2006) and worms (Ma et al. 2016; McCarroll et al. 2004) across a range of tissues from skin (highly proliferative) to brain (largely post-mitotic) (Glass et al. 2013; Hamatani et al. 2004; Kumar et al. 2013; Lu et al. 2004; Zahn et al. 2006), and is therefore not obviously related to a change in the requirement for mitochondrial biogenesis for proliferation. Reductions in mRNA level of mitochondrial proteins are often fairly small, but are clearly significant as recent proteomic studies in worms and mammals have discovered matching reductions in the ETC, ATP synthase, the tricarboxylic acid cycle and mitochondrial ribosomal proteins (Gomez-Serrano et al. 2017; Liang et al. 2014; Nishtala et al. 2013; Ori et al. 2015; Stauch et al. 2015; Walther et al. 2015; Xu et al. 2016).

This observation correlates well with reports that mitochondrial function decreases substantially with age (reviewed in Bratic and Larsson 2013) and notably, the downregulation is rapidly reversed in mice subject to health span-extending regimes including caloric restriction and metformin treatment (Kim et al. 2016b; Martin-Montalvo et al. 2013; Mercken et al. 2013; Whitaker et al. 2014). In addition, comparison of mice to long-lived naked mole-rats reveals that the latter possess higher levels of mRNAs encoding mitochondrial proteins (Yu et al. 2011). It has long been thought that reactive oxygen species (ROS) formed by mitochondrial respiration drive ageing through mutation and protein damage (Harman 1956); however, the strong positive correlation between mitochondrial protein gene expression and life/health-span implies high mitochondrial activity is associated with improved outcomes in ageing. This may result from the fact that mitochondrial ROS can also exert a beneficial effect through mitohormesis, in which low level oxidative stress induces a long lasting protective response that ultimately extends lifespan (reviewed in Yun and Finkel 2014), and/or that damaging levels of ROS can be produced in response to reduced mitochondrial activity or inefficient ETC assembly as observed in yeast and mice (Leadsham et al. 2013; Miwa et al. 2014).

There are some notable exceptions to this clear picture: firstly, ageing pathology in the *Drosophila* heart is driven by high expression of genes encoding mitochondrial proteins and can be ameliorated by local RNA interference against these mRNAs (Gill et al. 2015). Cardiac tissue has an unusually high energetic demand and very large numbers of mitochondria, so general effects of ageing on mitochondria may in this case be overridden by a tissue specific problem with high mitochondrial activity. Indeed, the opposite is observed for dietary restriction applied generally in *Drosophila*, which causes increased expression of ETC components while ETC inhibition reduces the lifespan extension bestowed by dietary restriction (Zid et al. 2009). Secondly, ETC gene expression is a negative predictor of lifespan in killifish and inhibition of the killifish ETC Complex I by rotenone increases lifespan (Baumgart et al. 2016). Again, this may stem from an unusually high mitochondrial activity in the short-lived killifish (we know of no comparative data to test this), but may also reflect the ability of low doses of rotenone to trigger lifespan extension through mitohormesis as previously reported in *C. elegans* (Schmeisser et al. 2013). It must also be considered that changes in ETC gene expression have a highly dose dependent effect of lifespan (Rea et al. 2007) and that accurate assembly of ETC complexes depends not only on protein concentration but also on stoichiometry (Miwa et al. 2014), so causal interpretations of these exceptional results will need to be very carefully validated.

Curiously, and in contrast to other studied eukaryotes, ETC mRNAs are upregulated with age in budding yeast (Laun et al. 2005 and our unpublished observations). An additional complication in this organism is the glucose repression system, which at a transcriptomic level serves to down-regulate the ETC and other respiration-related genes (Apweiler et al. 2012; Gancedo 1998). Early yeast gene expression studies suggested a shift away from glycolysis with age that is consistent with a loss of glucose repression (Kamei et al. 2014; Lin et al. 2001; Yiu et al. 2008). This would force an upregulation of ETC mRNAs acting in opposition to any underlying age-linked repression, providing a simple explanation for this apparent contradiction. Noticeably the only published study of cells aged in non-glucose media did not discover any upregulation of the ETC components relative to bulk mRNA despite possessing the statistical power to do so (Hu et al. 2014).

Overall, down-regulation of mRNAs encoding mitochondrial proteins appears to be a highly characteristic hallmark of ageing, one that has been observed in almost all published studies of ageing in multicellular eukaryotes. Although a few exceptions have been noted, these likely represent unusual and specialized properties of particular tissues or organisms.

### Downregulation of the protein synthesis machinery

Genes encoding components of the protein synthesis machinery are widely reported to be differentially expressed with age, in particular (though not exclusively) ribosomal proteins and ribosome biogenesis factors. Ribosome biogenesis is a highly conserved pathway involving a host of proteins that act primarily in the nucleolus to process the four ribosomal RNAs and mediate temporally and spatially coordinated binding of ribosomal proteins (reviewed in Venema and Tollervey 1999). Both translation and ribosome biogenesis are key targets of the mTOR pathway, which has dramatic effects on longevity (reviewed in Iadevaia et al. 2014; Lamming 2016), and it is therefore perhaps unsurprising that ribosome-related factors are so tightly regulated with age. The age-linked down-regulation of ribosomal proteins and ribosome biogenesis factors has been repeatedly noted in transcriptomic studies of budding yeast (Choi et al. 2017; Hu et al. 2014; Janssens et al. 2015; Kamei et al. 2014; Philipp et al. 2013; Wanichthanarak et al. 2015; Yiu et al. 2008). Given the high degree of conservation of these pathways and their apparent association with ageing, we expected similar observations to be almost ubiquitous in higher eukaryotes, but in fact, down-regulation of ribosome-related genes has been rarely reported in metazoan model organisms. We have seen one such report in *C. elegans* (Ma et al. 2016), two in *D. melanogaster* (Doroszuk et al. 2012; Pletcher et al. 2002), and one in mice that shows down-regulation only in some tissues (Zahn et al. 2007).

Why is this? Ribosomal proteins and ribosome biogenesis factors are very highly expressed, and changes in their expression that result in large differences in absolute number of mRNA molecules (and hence a substantial difference in absolute protein production) can represent small fold-changes (two-fold or less). This means that ribosome-related genes often miss differential gene expression cut-offs and fail to reach significance due to experimental and biological noise. Of course, these problems are addressed by improved statistical power, and it is reassuring that age-linked down-regulation of ribosomal protein and ribosome synthesis genes has been ubiquitously discovered by large cohort studies and meta-analyses in humans (Berchtold et al. 2008; Bryois et al. 2017; Kumar et al. 2013; Peters et al. 2015; Reynolds et al. 2015; van den Akker et al. 2014). Furthermore, although the age-related down-regulation may be small and rarely detected at an mRNA level in some organisms, effects on the proteome are striking, with progressive reductions in cytoplasmic ribosomal protein levels reported in worms and mice (Liang et al. 2014; Walther et al. 2015; Wilson et al. 2015).

It is tempting to link the down-regulation of genes encoding elements of the protein biosynthesis system simply to a reduction in activity of signalling pathways that promote proliferation (see Hallmark 4). This would imply that ribosome homeostasis is maintained in existing cells while fewer ribosomes are produced to supply new cells; however, the aforementioned reduction in ribosomal protein levels with age does not support this, and there have been many reports that translation efficiency decreases with age (reviewed in Rattan 1996). Indeed, comparison of gene expression data and microfluidic cell cycle monitoring in yeast show a progressive down-regulation of ribosomal protein mRNA from early age, which does not correlate at all to the observation that yeast maintain a well-synchronized cell-cycle progression for most of their life-span before a sharp reduction in division rate at the senescence entry point (Fehrmann et al. 2013; Janssens et al. 2015; Kamei et al. 2014). Furthermore, mRNA and protein levels for ribosomal proteins cannot be directly correlated as, at least in human cells, these proteins are constitutively over-expressed with excess being rapidly degraded through well-conserved mechanisms that maintain stoichiometry of ribosome components (Dephoure et al. 2014; Lam et al. 2007).

The down-regulation of ribosome biogenesis and ribosomal protein genes cannot be causal for ageing pathology as caloric restriction and rapamycin treatment both extend health/life-span but substantially decrease mRNA levels of ribosomal proteins through reduced mTOR activity (reviewed in Iadevaia et al. 2014). In fact, accumulation of ribosomal proteins is a predictor of shortened lifespan in yeast (Janssens et al. 2015; Janssens and Veenhoff 2016) and fibroblasts derived from Hutchinson–Gilford progeria syndrome (HGPS) patients show unusually high ribosome synthesis (Buchwalter and Hetzer 2017), while ribosomal proteins accumulated during ageing in *C. elegans* form aggregates that are likely be pathogenic (Reis-Rodrigues et al. 2012). Furthermore, low expression of ribosomal protein mRNA is predictive of longevity at both whole organism level in killifish, and when comparing long- and short-lived cell types in humans (Baumgart et al. 2016; Seim et al. 2016), while long-lived worm mutants routinely have reduced ribosomal RNA levels (Tiku et al. 2016). All this suggests that lower levels of ribosomal proteins improve health, and in support of this idea deletion of ribosomal proteins extend lifespan in budding yeast and *C. elegans* (Curran and Ruvkun 2007; Hansen et al. 2007; Kaeberlein et al. 2005; McCormick et al. 2015).

Together, these data suggest that down-regulation of ribosome components and ribosome biogenesis factors is a highly conserved hallmark of ageing, albeit one that can be hard to detect without robust experimental design and extensive replication. Many lines of evidence suggest that this down-regulation is a protective programme aimed at mitigating age-linked pathology. The cause of this decline is still mysterious however, and it is also unclear why organisms have not evolved to down-regulate ribosome biogenesis before the onset of any associated pathology.

### Dysregulation of immune system genes

The functional integrity of the immune system declines with increasing age, a phenomenon known as “immunosenescence” (reviewed in Weiskopf et al. 2009). This age-linked immune dysfunction is characterised by reduced response to pathogens as well as inappropriate over-activity. The latter is typified by “inflammaging”, a state of low-level chronic inflammation in the absence of infection observed in older people (reviewed in Franceschi and Campisi 2014). Several studies of ageing blood cell transcriptomes have identified significant changes in immune-related gene expression: genes associated with innate and adaptive immunity comprised the largest upregulated cluster in a meta-analysis of age-linked transcription changes in human blood (Peters et al. 2015) and inflammation-associated genes were found to be upregulated in mouse CD4+ and CD8+ T cells (Mirza et al. 2011). Observed transcriptional signatures of immune activation are not restricted to ageing blood: upregulation of inflammation-associated genes in the ageing brain has been reported in several studies (Berchtold et al. 2008; Lu et al. 2004), while expression of multiple inflammation genes correlated with age in human skin (Haustead et al. 2016). A meta-analysis of ageing gene expression changes in mouse liver found inflammation to be the most consistently upregulated pathway (Lee et al. 2012) and complement activation genes were found to be significantly upregulated in ageing muscle (Zahn et al. 2006). In such analyses, however, a large confounding variable is likely to be the changes in the repertoire of innate and adaptive immune cells that accompany immunological experience.

It is notable that dysregulation of immune-related genes extends to lower eukaryotes such as fruit flies, which lack adaptive immunity and possess only innate defences (Mylonakis and Aballay 2005). Transcription of immune response genes has consistently been reported to increase with age in *Drosophila* (Carlson et al. 2015; Doroszuk et al. 2012; Landis et al. 2012; Zou et al. 2000) and this has been shown to occur in multiple tissues (Girardot et al. 2006). In contrast with other organisms, *C. elegans* does not appear to display transcriptional signatures of immune activation with ageing; the *C. elegans* immune system is more primitive than that of *Drosophila*, which may explain its differing behaviour during ageing (Ermolaeva and Schumacher 2014). Alternatively, the failure to detect this may be technical; *C. elegans* immunity genes have very low basal expression, which may have impeded detection of significant changes on the microarray platforms used for most ageing transcriptomic analyses in worms. Similar to the adaptive immune system, the innate immune system is shaped by prior immunological experience in a process referred to as ‘trained immunity’ (Netea et al. 2016). Since immunological experience expands with age in normal environments, this may contribute to age-associated induction of inflammatory gene expression, even in animals lacking adaptive immunity.

Perhaps the clearest example of an age-related process intrinsically driving inflammatory gene expression, however, is the senescence associated secretory phenotype (SASP). Ageing is accompanied by an increase in the population of senescent cells, which have undergone an irreversible proliferative arrest in response to accumulated cellular damage, and this triggers the secretion of various cytokines, such as interleukin (IL)-6, IL-1α and IL-1β, chemokines, and proteases, which can promote inflammation (Coppe et al. 2010; Rao and Jackson 2016). Multiple types of damage are capable of inducing senescence in model fibroblast cultures, including telomere shortening, DNA damage and oncogene expression; each of these induction pathways is accompanied by specific gene expression changes (see for example Kuilman et al. 2008; Kural et al. 2016; Marthandan et al. 2016; Purcell et al. 2014), but are robustly associated with upregulated gene expression of mRNAs encoding cytokines and associated regulators, along with the expected down-regulation of replication and cell cycle genes (Contrepois et al. 2017; Kim et al. 2013; Kuilman et al. 2008; Lackner et al. 2014; Purcell et al. 2014). It is possible that transcriptional signatures of immune activation may reflect the increasing proportion of senescent cells with increasing age. Interrogation of upstream regulatory factors may provide insight into age-linked changes in immune-related gene expression. For example, NF-κB is a regulator of inflammation (Lawrence 2009) and SASP (Chien et al. 2011). A meta-analysis of gene expression microarray studies in a variety of cell types shows enrichment for NF-κB binding motifs in genes showing age-related changes in expression (Adler et al. 2007), and the same study found that inhibition of NF-κB activity in old skin cells reverses many age-induced gene expression changes. Conversely, constitutive NF-κB activity accelerates ageing in a mouse model (Jurk et al. 2014). Metformin treatment, an intervention that extends lifespan in various organisms, significantly reduces both NF-κB and chronic inflammation in old mice (Martin-Montalvo et al. 2013). Finally, basal NF-κB activity is upregulated with age in CD4+ T-lymphocytes (Bektas et al. 2014). Together, these studies suggest that NF-κB activity is a driver of increased inflammation during ageing.

In summary, there is substantial evidence supporting altered expression of immune-related genes as a transcriptional hallmark of cellular ageing. It is likely that these changes in gene expression are directly related to both increased inflammation and the general immune dysfunction that has been consistently observed during the ageing process.

### Reduction in growth factor signalling

Tight regulation of cell growth and division is essential for the development and survival of organisms and these processes are subject to the control of a vast network of intra- and extra-cellular signals (reviewed in Duronio and Xiong 2013). A number of studies report transcriptional signatures indicative of reduced cell growth and proliferation during ageing: expression of genes associated with cell growth is reduced in human and worm muscle tissue from older individuals (Ma et al. 2016; Zahn et al. 2006); genes associated with the cell cycle and DNA replication are downregulated in ageing mouse HSCs, CD4+ and CD8+ T-cells (Mirza et al. 2011; Sun et al. 2014); transcripts associated with the Growth Hormone/Insulin-like Growth Factor-1 (GH/IGF-1) signalling pathway are downregulated in aged mouse liver (Schumacher et al. 2008); while DNA methylation at the promoters of cell cycle genes increases in ageing mouse pancreatic β cells (Avrahami et al. 2015).

The effect of variation in growth factor activity on ageing is complex. Perturbations to insulin and insulin like growth factor signalling (IIS) extend lifespan and delay ageing pathology substantially in worms, flies, mice and humans (reviewed in Fontana et al. 2010). In contrast, there is evidence that reduced growth factor signalling in other contexts may lead to serious pathologies in ageing organisms. For example, vascular endothelial growth factor (VEGF), a factor required for skeletal muscle capillarization, is downregulated in old mice and humans (Ryan et al. 2006; Wagatsuma 2006). Expression of VEGF, as well as other growth factors such as TGF-β and FGF, showed a negative correlation with age in porcine fibroblasts (Vavken et al. 2010). Reduced angiogenesis—the formation of new blood vessels—is a well characterized ageing phenotype which likely contributes to the increased prevalence of cardiovascular disease in elderly patients (Lahteenvuo and Rosenzweig 2012). It is therefore possible that reduced growth factor signalling can have opposing effects on ageing and age-related pathology depending on the tissue-specific context.

The events that lead to downregulation of growth factor signalling during ageing remain unclear, although it is tempting to speculate that this hallmark may be linked to other cellular processes. It is possible for example, that reduced mitochondrial activity and protein synthesis could directly lead to inhibition of cell growth and proliferation due to a reduction in available energy and raw materials. Alternatively, reduction in growth and cell division signatures could be more representative of increasing prevalence of senescent cells in older individuals, and unsurprisingly, senescent cells of different cell types consistently display transcriptional profiles corresponding to cell-cycle arrest (see for example Shelton et al. 1999).

The mechanistic Target of Rapamycin (mTOR) pathway, which integrates nutrient availability and growth signalling, is ubiquitously associated with ageing in eukaryotes and mTOR inhibition by rapamycin has remarkable pro-longevity effects in disparate species (reviewed in Johnson et al. 2013). mTOR signalling stimulates translation and represses autophagy, both of which processes are tightly linked to ageing, however there is little evidence that mTOR signalling actually decreases with age and therefore it is unclear to what extent gene expression hallmarks of cellular ageing are driven by changing mTOR activity. Expression of genes regulated by downstream transcriptional effectors of mTOR has been found to decrease with age in blood and mesenchymal cells (Harries et al. 2012; Roforth et al. 2015), but this trend has not been reported in larger studies, and the impact of prolonged rapamycin treatment in higher eukaryotes is complex and does not correlate well with age-linked gene-expression changes (Fok et al. 2014a, b). Because mTOR acts so widely at a post-transcriptional level, it is likely that gene expression impacts of changing mTOR signalling will be indirect and hard to unambiguously identify. Furthermore, by far the strongest transcriptional effect of mTOR signalling is in regulation of RNA polymerase I and III activity, which produce transcripts that are not generally detected in steady state gene expression studies due to small product size, lack of polyadenylation and/or mapping issues (Kantidakis et al. 2010; Mayer et al. 2004; Tsang et al. 2010).

### Constitutive responses to stress and DNA damage

The existence of an age-linked stress response seems logical, particularly if ageing is accompanied by genetic and protein damage, and early transcriptomic studies in budding yeast reported evidence of this (Lesur and Campbell 2004; Yiu et al. 2008). However, the definition of stress was vague—gene expression changes were correlated to the Environmental Stress Response (ESR) defined by Gasch et al. (2000), which covers a huge range of genes that respond in broadly the same manner to a plethora of stresses or, it should be noted, simply changing carbon source. As such, the ESR seems more to represent the general response to slowing of growth rather than any defined stress response pathway, and encompasses specific elements seen in ageing, notably down-regulation of ribosome biogenesis, which may or may not be direct consequences of a stress response. Furthermore, these results have not been replicated in later, more comprehensive RNAseq studies making their significance unclear (Janssens et al. 2015; Kamei et al. 2014).

Much better evidence comes from *D. melanogaster*, where remarkable similarities have been noted between gene expression changes that occur during ageing and those that result from oxidative stress or hypoxia (Landis et al. 2004, 2012). Particularly, these encompass changes in heat shock proteins and proteases that might be expected to ameliorate the protein aggregation phenotypes associated with cellular ageing (reviewed in Josefson et al. 2017; Kim et al. 2016a; Labbadia and Morimoto 2015a). These changes appear to be positively correlated with lifespan in hormesis experiments as exposure of flies to a heat stress extends lifespan, with long-term expression differences between untreated and treated cohorts primarily lying in heat stress-response factors (Sarup et al. 2014). Similarly, studies of long-lived *Drosophila* cohorts have detected up-regulation of proteases and cytochrome p450 (Doroszuk et al. 2012; Sarup et al. 2011), while heat shock proteins also appear to be upregulated with age in worms as measured by both transcriptomic and proteomic methods (Lund et al. 2002; Walther et al. 2015). Complexities exist in the interpretation of mammalian studies as the GO terms for stress response overlap those covered by inflammation, and it is not always clear which process is truly changing. Various studies of mice have reported an age-linked upregulation of stress responses, particularly heat shock proteins that do not obviously stem from inflammation (Hamatani et al. 2004; Lee et al. 2000; White et al. 2015), however none of the large-scale human studies have reported any convincing age-linked stress response despite the widely reported increases in protein mis-folding in age-linked pathologies (reviewed in Hartl 2017). Fundamentally, many stress responses including the conserved integrated stress response act primarily at a post-transcriptional level, and are thus hard to detect in gene expression data. This is not to imply that there is no transcriptional aspect—indeed the integrated stress response activates many genes through transcription factor ATF4—but the transcriptional signatures of this have not been clearly detected in ageing gene expression datasets to our knowledge.

Even compared to other stress responses, evidence of increasing DNA repair is remarkably hard to find in transcriptomic studies. A handful of studies spanning a wide range of organisms report upregulation of repair genes (Etges et al. 2015; Haustead et al. 2016; Lesur and Campbell 2004; Lu et al. 2004; Peters et al. 2015) but most studies do not report any change, and a direct comparison of the ageing transcriptome with known gene expression responses to DNA damage in budding yeast revealed no significant overlap (Novarina et al. 2017). One of the two human studies that did detect upregulation of DNA repair factors was of skin samples and one of the two fly studies was of the desert dwelling *D. mojavensis* (Etges et al. 2015; Haustead et al. 2016), both expected to have high UV exposure across time which may well drive non-standard ageing responses, and we suggest that these are not representative. Furthermore, the genes identified in these studies do not identify any particular repair process, and other studies have actually reported down-regulation of DNA repair genes (Hamatani et al. 2004; Sun et al. 2014). Based on the sparsity of evidence, we therefore do not consider upregulation of genes encoding DNA repair factors to be a general feature of the ageing transcriptome. However, it is important to consider that this represents a failure to find an enrichment of DNA repair as a functional category amongst genes with altered expression across ageing. In contrast, very clear general effects of DNA damage on gene expression have been reported. As normal mice age, long genes become downregulated more than short genes, a phenomenon that is much stronger in *Ercc1*^Δ/−^ repair deficient mutants, suggesting that transcription-blocking legions accumulate with age and bias the transcriptome (Vermeij et al. 2016). Similarly, evidence that direct damage to promoter sequences reduces gene expression in neurons has also been reported (Lu et al. 2004).

Overall, good evidence exists for age-associated occurrence of both protein and DNA damage (Lourenco dos Santos et al. 2015; Lu et al. 2004; Tsakiri et al. 2013; Vermeij et al. 2016), and responding to this damage often enhances longevity, but it appears that few organisms constitutively upregulate repair during ageing, and only in a subset of tissues. This may reflect the argument that repairing somatic damage is expensive, and is likely only performed when it would otherwise impact reproduction (Kirkwood 1977). As such, although DNA damage and defective protein folding are tightly associated with the progression of ageing pathology, transcriptional responses to these stresses are weak and must remain a rather questionable hallmark of the ageing process—informative when present but not indicative of youth or healthy ageing when absent.

### Dysregulation of gene expression and mRNA processing

There is evidence that the regulation of gene expression itself is affected by ageing, leading to intercellular heterogeneity in mRNA content that may represent noise. Bahar et al. (2006) tested for the presence of noise directly by assaying the expression level of a panel of genes in single cells isolated from mouse cardiac tissue. They found increased heterogeneity in the expression of both housekeeping and heart-specific genes in cells from older mice, suggesting a general defect in transcriptional regulation. Such heterogeneity may arise over considerable time in long-lived cardiac cells, but remarkable heterogeneity was also observed within days of induction of senescence in cultured fibroblasts relative to quiescent controls (Wiley et al. 2017). A recent study expanding this approach to the entire transcriptome using single cell RNAseq found that transcriptional heterogeneity increases in CD4+ T cells from older mice upon immune stimulation (Martinez-Jimenez et al. 2017). Increased heterogeneity of transcription has also been observed on the organismal level: Rangaraju et al. (2015) found that genes sharing the same functional group show opposing changes in transcript abundance in ageing worms, a phenomenon they referred to as “transcriptional drift”. Attenuation of transcriptional drift by inhibiting serotonergic signalling resulted in delayed physiological decline and increased lifespan. A similar process of transcriptional drift has been observed in ageing mice (Southworth et al. 2009).

Increasing transcriptional heterogeneity and noise may be underpinned by changes in chromatin structure that occur during the ageing process. Promoter unmasking through progressive loss of histones with age is a major driver of gene expression noise in yeast (Hu et al. 2014), and replicative ageing similarly depletes histones in mammalian cells (O’Sullivan et al. 2010) and even in mice (Kannan et al. 2016). The trimethylation of histone H3 at lysine 36 (H3K36me3) is a histone modification associated with the bodies of actively transcribed genes that repress cryptic transcription initiation (Butler and Dent 2012). Sen et al. (2015) found that H3 K36me3 levels decrease at a number of loci in ageing yeast cells, and these loci show an age-induced increase in cryptic transcription. Loss of the K36me2/3 demethylase Rph1 counteracted both the loss of H3 K36me3 and increased cryptic transcription at these genes and extended lifespan. H3K36me3 enrichment is also anti-correlated with the magnitude of gene expression change in ageing in worms and flies (Pu et al. 2015). Therefore, a recurring theme of ageing throughout eukaryotes is a generalized loss of gene silencing on the level of chromatin.

Defects in nuclear architecture and particularly in heterochromatin are associated with progerias including HGPS and Werner syndrome (Scaffidi and Misteli 2006; Shumaker et al. 2006; Zhang et al. 2015). Euchromatinisation of tightly repressed regions is likely to have serious consequences for the cell, exemplified by the observation that transposable elements become active in the ageing fly brain and mouse liver (De Cecco et al. 2013b; Li et al. 2013), and non-coding RNAs are transcribed from normally silent pericentromeric repeats in HGPS cells (Shumaker et al. 2006). Large-scale changes in chromatin structure are also a feature of cellular senescence. Senescent cells harbour cytologically visible clusters of heterochromatinised DNA termed senescence-associated heterochromatin foci (SAHFs) (Narita et al. 2003). Single-cell bisulphite sequencing revealed widespread hypomethylation with foci of hypermethylation in senescent human myofibroblasts (Cruickshanks et al. 2013). Loss of silencing of heterochromatic regions in senescent human fibroblasts leads to transcription and mobilization of retroelements, demonstrating an important link between senescence-associated changes in the chromatin landscape and dysregulation of gene expression (De Cecco et al. 2013a). Loss of silencing can therefore be considered a conserved hallmark of ageing, but whereas this leads to a widespread upregulation of gene expression in yeast the effect is more subtle in higher eukaryotes, resulting in loss of repressive activities and associated expression of normally silenced transcripts both from heterochromatic and euchromatic domains.

Changes in post-transcriptional processing of mRNA present another potential source of increased transcriptional variability. An early study reported a three-fold decrease in the proportion of cytosolic RNA that is polyadenylated in the livers of old rats compared with younger individuals (Yannarell et al. 1977). More recently, reanalysis of microarray data from human peripheral blood leucocytes in 698 individuals concluded that the pathways most disrupted with ageing involve genes associated with messenger RNA splicing, polyadenylation and other post-transcriptional events (Harries et al. 2011). Consistent with this result, a later microarray study of blood samples in two human populations found age-linked changes in the transcript levels of approximately one third of all splicing factors (Holly et al. 2013a). Expression of splicing factors in these blood samples was found to be correlated with that of the DNA damage response kinase Ataxia Telangiectasia Mutated (ATM). The authors also observed changes in splicing factor expression in senescent primary fibroblasts and endothelial cells and showed that RNAi knockdown of ATM resulted in upregulation of a subset of age-responsive splicing factors. A follow-up study from the same group found an association between the expression of a number of splicing factors and lifespan in several tissues across six different mouse strains and between expression of the splicing factors HNRNPA1 and HNRNPA2B1 and longevity in humans (Lee et al. 2016). Perhaps the most detailed link between splicing homeostasis and longevity has been demonstrated in *C. elegans.* A recent study identified global defects in pre-mRNA splicing as a feature of ageing in *C. elegans*, and showed that the worm homologue of splicing factor 1, SFA-1, was required for lifespan extension by dietary restriction and modulation of the TORC1 pathway (Heintz et al. 2017). Furthermore, overexpression of SFA-1 was found to be sufficient for lifespan extension. Aside from contributing to transcriptional noise, defects in splicing and pre-mRNA processing in general may contribute to aging pathologies either through retention of non-coding regions in mRNA or disruption in the pattern of exon usage in alternatively spliced transcripts. Evidence for the latter was reported in one study that found that 0.3–3.2% of genes across several tissues become alternatively spliced in aging male mice (Rodriguez et al. 2016), although it is not clear whether this is a direct consequence of splicing defects or rather represents a series of cellular responses to the effects of ageing. Furthermore, the same study found that alternative splicing was substantially altered in HGPS skin cells. However, only a handful of ageing transcriptome studies provide information on changes to post-transcriptional processing, and further studies will be required to determine the extent to which such changes contribute to the ageing process.

In summary, gene expression de-regulation represents a distinct transcriptional hallmark of cellular ageing that is likely to be directly associated with highly conserved age-induced changes in chromatin structure.

## Relation to the hallmarks of ageing

We compiled our list of hallmarks without direct comparison to the published hallmarks of ageing (Lopez-Otin et al. 2013), but reassuringly there is substantial concordance between the two sets. Some of the gene expression hallmarks correlate very well to the general hallmarks of ageing: down-regulation of mitochondrial protein gene expression will very likely contribute to the *Mitochondrial dysfunction* hallmark of ageing, particularly given that upregulation of these genes is caused by many lifespan-extending treatments. Similarly, the impacts of *Cellular senescence* and *Altered intracellular communication* are well represented by dysregulation of immune system genes, consistent with this being perhaps the most prominent and reproducible phenotype of ageing in mammals.

In contrast, the *Loss of proteostasis* hallmark is poorly reflected in gene expression datasets. As defined, this hallmark encompasses chaperones and responses to general protein mis-folding and damage for which there is limited evidence of change in the transcriptome, along with proteolytic pathways and autophagy that have only sporadically featured in reports of differential gene expression. It should be noted that changes in the inducibility of chaperones and damage response factors is a prominent feature of ageing in some and potentially all eukaryotes (see for example Labbadia and Morimoto 2015b), but are not factored into our definition of gene expression hallmarks as the vast majority of ageing transcriptome studies overlap only in basal gene expression analysis and we therefore cannot address how cells of different ages respond to environmental challenges.

Similarly, it is not clear which, if any, of the hallmarks of ageing can be directly attributed to the down-regulation of protein synthesis factors that is so clearly detected at the gene expression and proteomic levels. It may be that this is an effect rather than a cause; *Deregulated Nutrient Sensing* clearly relates to the gene expression impacts of reduced growth factor signalling, but may also be the driver of reduced expression of protein synthesis factors given the tight regulation of ribosome biogenesis by nutrient signalling complexes such as TORC1. Nonetheless, the lifespan extension caused by deletion of ribosomal proteins in lower eukaryotes suggests that down-regulation of protein synthesis is more than a passive biomarker.

Two hallmarks of ageing that might be expected to instigate a strong gene expression response are *Genomic instability* and *Telomere attrition*, however as discussed above, we see little evidence of a constitutive transcriptional response to DNA damage in ageing cells. The effects may well be indirect however; telomere attrition is the classic pathway to senescence and therefore telomere attrition may well contribute to the immune dysfunction gene expression hallmark. The de-differentiation and increased transcriptional noise observed in the transcriptome probably result from a combination of *Genomic instability* and *Epigenetic alterations*, which will have stochastic effects on gene expression at the individual cell level.

## Outlook

The identification of gene expression hallmarks associated with the cellular ageing process has multiple applications, despite the technical difficulties inherent in their detection. On one hand, characteristic patterns of age-linked gene expression change provide useful insights into the underlying drivers of ageing in diverse organisms, and will be very helpful in identifying outlier systems that do not reflect the normal progress of ageing. Furthermore, we envisage that gene expression differences will substantially precede ageing pathology, and therefore have significant potential as ageing biomarkers. Although substantial power is required to detect the hallmarks, this is not necessarily a problem given the rapidly decreasing cost of transcriptomic analysis, and although transcriptomic markers are unlikely to outperform well validated protein markers in studies focused on ageing, the generality of transcriptome analysis across studies in a broad range of fields should allow many additional datasets to be analysed for information on the ageing process. Discovering interventions that retard the ageing process as a whole is obviously a primary goal, and the ability to screen for beneficial effects in reduced timescales by transcriptomics should be extremely beneficial. Similar arguments can of course be made for the use of epigenetic ageing clocks, and we consider that the best approaches will integrate epigenetic and transcriptomic information, given that both are amenable to large studies.

Benefits should not be restricted to purpose-made pharmaceutical studies and can also address lifestyle impacts on the ageing process. For example, it has long been known that dietary restriction is able to mitigate the physiological decline associated with ageing and extend lifespan to a significant extent. However, it is not clear that general dietary restriction is absolutely required to gain these benefits, with recent research in *Drosophila* showing that calorie content is not the primary driver of ageing and that extended lifespan can be combined with normal fecundity through appropriate choice of dietary amino acid composition (Mair et al. 2005; Piper et al. 2017). Furthermore, we have recently demonstrated that yeast aged on a non-standard but non-restricted diet maintain high fitness throughout life (Frenk et al. 2017), showing that novel dietary interventions can substantially retard the ageing process. Vast transcriptomic datasets are likely to be collected by the next generation of population-wide human studies, which could plausibly be screened for differences in ageing hallmarks then cross correlated to lifestyle information. More specific information on the impact of diet is also likely to be attained by analysing the burgeoning nutrigenomics datasets.

Transcriptomic analysis has clear limitations (see “Additional Information” section); gene expression differences at the mRNA level are not completely descriptive of the state of the proteome, let alone the metabolome, and a comprehensive understanding of ageing requires these also to be studied. However, transcriptomic datasets are well suited to large-scale acquisition and analysis, and any insights into the contributions of more nuanced aspects of diet concerning lifelong health gleaned from them would be of great importance for informing public health messages. More broadly, the identification of gene expression hallmarks associated with the ageing process across a wide-range of organisms reveals conserved aspects of the ageing process that should be of general use in discovering critical underlying mechanisms of cellular ageing.

## Additional Information: What does a change in gene expression mean?

It is important to consider the real impacts on the cell and the organism of a change in mRNA abundance relative to a control, the actual quantity measured in a transcriptomic experiment. The absolute abundance of an mRNA is not easy to measure and has to be normalised to something else (for example total read count in an RNAseq library), and when many highly expressed genes change in expression this can distort the measurements. For example, in yeast all mRNAs accumulate with age, and therefore reported mRNA down regulation in fact represents slower accumulation relative to average, a difference that is only detectable with appropriate normalisation for cell number (Hu et al. 2014). Similar effects may well distort measurements of age-linked gene expression change in higher eukaryotic cells as ageing impacts many highly expressed housekeeping genes that make up a substantial fraction of the transcriptome.

mRNA levels are determined by rate of mRNA synthesis balanced by rate of degradation and/or mRNA loss through cell division, and are therefore not a direct measure of gene expression. Furthermore, the effect of increased mRNA level is very much dependent on ribosome availability, with a limited pool of ribosomes needing to prioritise translation and providing an unequal representation in the nascent proteome of mRNA abundance. The protein complement of the cell is further controlled by targeted proteolysis and autophagy, meaning overall that mRNA levels provide at best a fairly crude insight into the proteome beyond a binary read-out of whether a gene is expressed or not, particularly in slowly- or non-dividing cells where a delicate homeostasis exists between protein synthesis and degradation.

Interpretation of gene expression data alone can therefore be misleading. For example, in yeast ribosomal proteins accumulate with age relative to other proteins despite the repeated observation that ribosomal protein mRNA levels decrease relative to the transcriptome average (Hu et al. 2014; Janssens et al. 2015). This reveals a constitutive imbalance between ribosome synthesis and degradation. In contrast, the ribosomal protein content of longer lived organisms decreases with age (Liang et al. 2014; Walther et al. 2015; Wilson et al. 2015), as does mRNA abundance, suggesting that degradation rates are matched to synthesis in young cells and that as ribosomal protein gene expression decreases with age the degradation rates are constant (which has been confirmed experimentally in mouse liver Karunadharma et al. 2015). Therefore, the same trend in age-linked mRNA abundance in yeast and higher eukaryotes has differing effects at the protein level. In contrast, changes in gene expression can be masked at the protein level; although a reduction in ribosomal protein gene expression is also caused by mTOR inhibition under caloric restriction, this is balanced by a large extension of protein half-life in mice, and therefore a reduction in translation efficiency is not expected (Karunadharma et al. 2015).

Overall, gene expression data must be treated as an insight into, not a measurement of the physiology of the cell. Changes in mRNA abundance are to some extent informative about protein production, but more usefully provide an insight into which proteins are being prioritised for maintenance and what reactive mechanisms are being induced in response to intrinsic and extrinsic changes. The expression of housekeeping genes such as ribosomal and ETC components would be examples of the former, whereas stress response and inflammation-related genes would be in the latter category although this distinction is not of course absolute.

We suggest that six hallmarks encompass the conserved gene expression changes associated with normal ageing: downregulation of genes encoding mitochondrial proteins, downregulation of the protein synthesis machinery, dysregulation of immune system genes, reduction in growth factor signalling, constitutive responses to stress and DNA damage, and dysregulation of gene expression and mRNA processing.
